# Influences of a Luck Game on Offers in Ultimatum and Dictator Games: Is There a Mediation of Emotions?

**DOI:** 10.3389/fpsyg.2020.00013

**Published:** 2020-02-03

**Authors:** Olimpia Matarazzo, Barbara Pizzini, Claudia Greco

**Affiliations:** Department of Psychology, Università della Campania Luigi Vanvitelli, Caserta, Italy

**Keywords:** ultimatum game, dictator game, offer, contextual factors, emotions

## Abstract

The ultimatum (UG) and dictator (DG) games are two tasks where a sum of money has to be divided between two players: a proposer and a receiver. Following the rational choice theory, proposers should offer the minimum in the UG and nothing in the DG, due to the presence/absence of the receivers’ bargaining power. The fact that people generally make non-negligible offers in both games has suggested divergent explicative hypotheses and has generated extensive research to examine exogenous and endogenous factors underlying such decisions. Among the contextual factors affecting the proposers’ offers, the sense of entitlement or of ownership has been shown to reduce offers significantly. A frequent way to induce the sense of entitlement/ownership has been to assign the role of proposer to the player who apparently has better scored in skill tasks executed before the UG or DG or has more contributed, through a previous luck game, to the amount to be shared. Such manipulations, however, could produce a possible overlapping between “ownership” and “merit,” that in this study we aimed to disentangle. We manipulated the participants’ initial endowment through a luck game, by increasing, decreasing or leaving it unchanged, to investigate whether winnings or losses by chance influenced offers in UG and DG in similar or different ways depending on their respective features. All participants played as proposers but this role was apparently random and disconnected from the outcomes of the luck game. Furthermore, we investigated whether the putative effect of experimental manipulation was mediated by the changes in emotions elicited by the luck game and/or by the emotions and beliefs related to decision-making. We used a non-economic version of the games, in which tokens were divided instead of money. In the study, 300 unpaid undergraduates (*M* = 152) from different degree programs, aged between 18 and 42 years, participated. The results revealed that the effect of outcome manipulation on offers was moderated by the specific structure of the UG and DG. Instead, emotional reactions barely mediated the effect of the experimental manipulation, suggesting that their role in those decisions is less relevant than is assumed in the literature.

## Introduction

The ultimatum game (UG – Güth et al., 1982) and the dictator game (DG – Kahneman et al., 1986; Forsythe et al., 1994) are two tasks that are widely used to investigate people’s behavior in strategic interactions, i.e., the situations where at least two persons are involved in decision-making (von Neumann and Morgenstern, 1944; Camerer, 2003). Thus, decision-makers have to consider others’ expectations and preferences when choosing the course of action.

In the UG, two players, a proposer and a receiver, have to divide a sum of money provided by the experimenter. The proposer has to decide how much to offer to the receiver, who can either accept or reject the proposal, knowing that if she rejects it, both players will earn nothing. According to the predictions of rational choice theory (von Neumann and Morgenstern, 1944), the proposer should offer the minimum possible amount and the receiver should accept any proposal, since any sum is better than zero.

However, numerous studies conducted in the fields of experimental economics and psychology have demonstrated that both players systematically disregard these predictions and tend to behave fairly. Indeed, in the UG, most proposers offer between 40 and 50% of the total amount, while receivers tend to reject offers below 20 or 30% (for reviews, see Camerer, 2003; Güth and Kocher, 2014).

Regarding the proposers’ behavior, which is the topic of this paper, two main hypotheses have been advanced: one is represented by “social preference models” (e.g., Fehr and Schmidt, 1999, 2006; Bolton and Ockenfels, 2000; Falk and Fischbacher, 2006), according to which positive offers are essentially driven by a sense of fairness or inequity aversion. The opposite position claims that people are motivated by self-interest and that fair offers are strategic means to avoid rejection (e.g., Pillutla and Murnighan, 1995, 2003; Fellner and Güth, 2003; Ding et al., 2014; Chen et al., 2017), a form of behavior that has also been considered as an expression of risk aversion (Holt and Laury, 2002; Karagözoğlu and Urhan, 2016).

To disentangle the motivations underlying the proposers’ behavior, the DG, a game created to model charitable giving, has been frequently used. In this game, the role of proposers (or allocators) is unmodified, but receivers cannot reject any offer, and thus have no decisional power. The studies on the DG showed that, on average, allocators give 25% of their endowment and that only about a third of them offer nothing, while about 17% choose an even split (see the meta-analysis of Engel, 2011). However, the difference between the offer amounts in the UG and the DG can be interpreted as evidence in favor of both theoretical viewpoints by stressing either the offers’ decrease or their persistence.

The studies directly comparing the UG and DG have obtained divergent results: many of them (e.g., Mellers et al., 2010; Baumert et al., 2014; Bechler et al., 2015) reported higher offers in the UG compared to the DG, corroborating the hypothesis that proposers’ behavior is driven by strategic motives due to possible rejections. Conversely, other studies (van Dijk and Vermunt, 2000; Handgraaf et al., 2008), which have used both the UG and DG or the so-called delta UG (Suleiman, 1996), in which the receivers’ bargaining power varied along a continuum from 1 (=UG) to 0 (=DG), have found higher offers in the DG than in the UG. According to the authors, proposers tend to employ two different criteria in making offers: strategic reasons, when recipients have bargaining power, and social responsibility, when recipients are powerless.

Independently from the different offer amounts between the UG and the DG, there are several factors affecting the proposers’ offer in both games. These include individual differences in selfishness or altruism (van Dijk et al., 2004; Baumert et al., 2014); the size of the amount to divide, according to which offers decrease as the amount size increases, at least for the DG (see the meta-analysis of Larney et al., 2019); the social distance between the players, which leads offers to diminish when the distance increases (Hoffman et al., 1996; Charness and Gneezy, 2008; Rachlin and Jones, 2010; Bechler et al., 2015); the level of anonymity in the experimental setup, according to which offers decrease when anonymity is most assured (Hoffman et al., 1994; Charness and Gneezy, 2008; Franzen and Pointner, 2012); and the sense of entitlement or of ownership – i.e., the sense of having more merit or more property rights than the other player – according to which offers decrease when proposers feel entitled to have more. The sense of entitlement/ownership (the two terms being often used interchangeably) has been manipulated by varying the initial ownership of property between proposers and receivers in the UG (e.g., Leliveld et al., 2008) or through a two-stage paradigm, in which the amount to share in the UG or DG, or the assignation of the players’ roles stems from previously manipulated skill tasks in the first stage (e.g., Hoffman et al., 1994; Schotter et al., 1996; Cherry et al., 2002; Cappelen et al., 2007; Oxoby and Spraggon, 2008; Korenok et al., 2017). In a few studies on UG, the sense of entitlement/ownership has also been manipulated by using luck games, which do not involve any personal ability, in the first-stage. The results have shown that gaining by chance at least part of the amount size to subsequently share (van Swol and Braun, 2014), or contributing more than the other player to the common budget to be divided (Bland et al., 2017) leads proposers to make lower offers.

However, a study that has manipulated the outcomes of a luck game, through which the initial endowment to be shared in the UG increased, decreased or remained unchanged (Matarazzo et al., 2017), has found no evidence for the hypothesis that increasing one’s original endowment before the UG should increase the sense of ownership and thus should decrease offers. Indeed, offers decreased after a negative outcome compared to the positive or unchanged outcomes. Presumably, after a loss, participants perceived themselves as “poorer” than before the luck game and thus felt they were authorized to make smaller offers. Therefore, the manipulated luck game seems to change the perception of the amount size available for offers, which decreases after a loss, though it does not vary after a winning.

To our knowledge, no other study has so far jointly investigated the effects of an augmentation or diminution of the initial endowment on the proposers’ offers. Thus, the issue whether changes due to chance of one’s initial endowment modify the reference point for offers (and through which mechanisms) deserves to be further investigated, also for the DG. Indeed, the structural differences between the UG and DG could interact with the effects of the luck game manipulation by inducing divergent mechanisms for each of them, as some studies (van Dijk and Vermunt, 2000; Handgraaf et al., 2008) have shown.

The role of emotions in proposers’ decisions is another topic that needs to be more deeply investigated. The fact that emotion plays a central role in informing and motivating decision-making is widely acknowledged (for a review, see Lerner et al., 2015). However, with regard to economic games, most studies have been conducted on emotional reactions underlying the receivers’ rejection of unfair offers (e.g., Pillutla and Murnighan, 1996; Sanfey et al., 2003; van’tWout et al., 2006; Moretti and di Pellegrino, 2010). As for the proposers, fear of rejection has been broadly considered as at least partially responsible for the ostensibly fair offers in the UG, although this emotion has often been inferred from the proposers’ behavior rather than directly assessed (but see Leliveld et al., 2008 for its direct assessment). The same considerations are extensible also to empathy and sense of fairness, which are assumed to increase offers in both the UG and DG. Only a few studies have examined the effect of previous mood or emotions on offers in the UG and DG (e.g., Mellers et al., 2010, experiment 3; Forgas and Tan, 2013; Matarazzo et al., 2017), and their findings are discordant. Mellers et al. (2010, experiment 3) found that a positive incidental affect increased cooperation, whereas Forgas and Tan (2013) found the opposite results. Matarazzo et al. (2017) found mixed evidence.

## Overview

In the present study, by using a two-stage paradigm, we examine whether a manipulated (positive/negative/unchanged) outcome obtained in a luck game influences the proposers’ decisions in the UG and DG in similar or different ways, depending on the structural features of each game.

We assume that the outcomes of a luck game should modify the reference point for offers: compared to the unchanged outcome, winning or losing something should induce in the participants the perception of being “richer” or “poorer” than before the luck game and thus offers should be made, rather than on the current endowment, on the difference between previous endowment and the current one.

Starting from this supposition, some contrasting hypotheses are tested. According to the hypothesis based on the sense of ownership – i.e., winning something makes winners believe they have property rights on that (van Swol and Braun, 2014; Bland et al., 2017) – a positive outcome, compared to the control condition and/or to a negative outcome, should reduce offers in both games, especially in the DG, due to the receivers’ powerlessness. Thus, two main effects are expected in conformity with this hypothesis, one due to the experimental manipulation and the other due to the type of game.

According to the hypothesis based on the assumption that people have intrinsic concerns for fairness and altruism (Fehr and Schmidt, 1999, 2006), a positive outcome, due to good luck (i.e., without merit), should increase offers in both games, without a significant difference between them. Put it in other terms, if people have a sense of fairness or inequity aversion, they should behave more generously (i.e., should offer more) when they have more. Since in the positive outcome condition, participants almost doubled their initial endowment, they should feel “richer” than before the luck game and, thus, they should be more willing to share their “luckiness” with receivers. Consequently, offers should increase compared to the control condition, where the endowment was unchanged after the luck game. Note that a decrease of offers in both UG and DG after a negative outcome is compatible with this hypothesis. Having less than before could authorize people to make reduced offers.

Conversely, an increase of offers with a negative outcome would be hardly understandable in the DG, while in the UG it could be due to the desire of keeping at least a part of the remaining endowment: consequently, higher offers should be motivated by the desire of avoiding rejection and could be viewed as an expression of risk aversion (Holt and Laury, 2002; Karagözoğlu and Urhan, 2016).

A third hypothesis is that the structural differences between the UG and DG would lead proposers to behave in opposite ways, i.e., in conformity with strategic concerns in the UG, where receivers have bargaining power, and in conformity with a sense of fairness or of social responsibility in the DG, where receivers are powerless (van Dijk and Vermunt, 2000; Handgraaf et al., 2008).

In all experimental conditions, the role of the proposer is apparently assigned randomly to one of the two participants who played a similar luck game separately from each other, though respective results are not communicated. However, at the end of the luck game, the instructions specify that both players have a certain endowment, and that the player who will have the role of proposer has to share his/her endowment according to the rules of the UG or DG. This information has a double function: the first is to make the random assignment of the role of proposer believable, thus avoiding, in the condition of positive outcome, the impression that the player who gained something at the luck game deserved the role of proposer.

The second function is to investigate whether the uncertainty about the endowment of the receiver affects offers (see Engel and Goerg, 2018 for findings in the DG showing that this uncertainty tends to increase offers). Actually, we expect that, regardless of the type of hypothesis tested, to know that receivers have already some endowment, though of unknown size, should reduce offers in both games, compared to those reported in the literature. Indeed, this information should reduce both generosity (since receivers are not needy) and fear of rejection in the proposers (since receivers, having already something, should be more willing to accept even small offers and less willing to punish proposers by rejecting their offers and leaving them without anything).

The second aim of the study is to investigate whether the putative effect of the luck game manipulation on offers is mediated by the changes in emotions elicited by the luck game (EMs) and/or by the emotions and beliefs (EBs) related to decision-making. Among the latter, we included fear of rejection, empathy, and a sense of fairness that in the literature are deemed to underlie the decisions in the UG and/or DG but which, to our knowledge, have not been directly evaluated by the participants with the exception of Mellers et al. (2010) for fairness. The EMs related to positive/negative outcomes, which we took into account were: disappointment, feeling lucky, happiness, irritation, sadness, and satisfaction. We chose to consider specific emotions instead of mood or global affect because we assumed, in conformity with the appraisal theories (Frijda, 1986; Lazarus, 1991; Lerner and Keltner, 2000; Roseman, 2008), that specific emotions, by encompassing in their meaning the appraisal of the eliciting events, entailed more semantic information than valenced (positive/negative) emotional states, such affect or mood.

In detail, we assume that the experimental manipulation should affect the intensity of the six EMs, self-assessed before and after the luck game, and the intensity of a set of EBs, which in the literature are assumed to underlie decision-making about the offer in the UG or DG (see “Materials and Procedure” section). In turn, changes in EMs and in EBs intensity should affect offers, transmitting the putative effect of the independent variable on the dependent one, i.e., mediating its effect. If EMs and EBs act as mediation variables, it is possible to infer that the process through which contextual factors, such as a previous luck game, influence decision-making about offers is at least partially based on emotional reactions and/or on emotionally charged beliefs. As we said, to our knowledge, no studies have tested this hypothesis. However, EBs can have a direct effect on decision-making, without having a mediation effect, i.e., they can be elicited by the features *per se* of the two games and not by the experimental manipulation of the contextual factors. Thus, they can influence decision-making about offers regardless of the context in which UG or DG are played. In this case, one should infer that UG and/or DG entail emotionally charged beliefs to which the decision about offer could be at least partially due.

We used non-economic versions of both the UG and DG, in line with a few previous studies (Ciampaglia et al., 2014; Matarazzo et al., 2017), whose results suggest that proposers’ decisions in these conditions are similar to those found in the economic versions. Moreover, participants were unpaid volunteer. Thus, we did not offer either a show-up fee or a performance fee to participants. Although monetary incentives are widely used in the literature on experimental economics (for a review, see Hertwig and Ortmann, 2001), we based our choice on the work of those authors in the fields of experimental economics or psychology, which point out that monetary incentives are not necessary and may even be detrimental to evaluate how people behave in hypothetical contexts, such as decision-making (e.g., Kühberger, 2001; Read, 2005; Locey et al., 2011).

## Materials and Methods

### Design

A 2 (game: UG vs. DG) × 3 (outcome: unchanged/positive/negative outcome) between-subject design was created. The different outcomes were obtained by manipulating the results of a luck game (a card game) played in the first part of the experiment, before playing the UG or the DG.

### Participants

In this study, 300 unpaid undergraduates (*M* = 152) from different degree programs, aged between 18 and 42 (*M* = 22.20; SD = 3.029), participated: 150 played the UG and 150 the DG. They were randomly assigned to the six experimental conditions.

The participants were recruited two at a time; attention was paid to avoid any contact between them to ensure mutual anonymity. They were settled in two nearby but separate rooms and were told that they would interact via the Internet in the second part of the experiment. Actually, there was no real interaction between them. To preserve anonymity, it was specified that they would not encounter each other even after the experiment. All participants executed the tasks individually, without the presence of the experimenter. Before the experiment, participants gave their informed consent.

### Materials and Procedure

The experiment was implemented on “E-Prime 2.0” software. Its feasibility had been tested through a pilot study in which 30 unpaid students had participated. It revealed that the instructions were clear and that participants expressed no suspicion about the interaction with the other participant or about the outcome’s manipulation.

In the general instructions, the participants were informed that the study was divided into two parts. In the first part, they would have to play a simple luck card game separately from each other. Concerning the second part, the instructions differed depending on the type of game. In the case of the UG, participants were told that in the second part they would have to make a decision “by interacting with the other participant.” In the case of the DG, they were told that either themselves or the other participant would have to make a decision that would affect both participants.

To play the luck game, participants were provided with a certain number of tokens which varied as a function of the experimental condition: the participants in the unchanged outcome had 19 tokens, those in the positive outcome had 10 tokens, those in the negative outcome had 40 tokens. The luck game consisted of eight draws from a deck of 40 cards: with each draw, some tokens could be won or lost, according with the rules presented in the game description (e.g., if you draw 8 or 10, you gain 5 tokens; if you draw 2 or 4, you lose 4 tokens, etc.). The result of the luck game was manipulated so that, at the end of the eight draws, all participants had 19 tokens. Note that those in the positive outcome almost doubled the initial number of tokens, whereas those in the negative outcome lost almost half of it. We chose to provide proposers with an odd rather than an even number of tokens to prevent us from misinterpreting the choice of the simplest option, namely splitting the tokens in half, as a fair choice.

To check whether the experimental manipulation affected the participants’ emotional state, they were asked to assess the intensity of six emotions – disappointment, feeling lucky, happiness, irritation, sadness, and satisfaction – before and after playing the card game, on a 9-point Likert scale (1 = not at all; 9 = extremely). The emotions’ presentation order was randomized.

Afterward participants were told that, after the luck game, both of them had a certain number of tokens: thus, both of them could participate in the second part of the experiment. However, none of them knew the number of tokens of the other participant.

Then, a single session of the UG or DG was played. The instructions specified that one of the two participants, extracted at random, had to decide whether and how to divide his/her available budget (19 tokens) with the other participant, according to the rules of the UG or the DG. Actually, all participants played the role of proposer. Reaction times during decision-making were registered. After making their offer, all participants were asked to assess, on a 9-point Likert scale (1 = not at all; 9 = extremely), the intensity with which they had experienced a set of EBs, which in the literature are assumed to underlie decision-making about the offer. They were randomly presented and slightly diversified depending on the principal game. In Table 1, EBs were reported as a function of the type of game.

**TABLE 1:** Emotions and Beliefs assessed after the decision about the offer in the UG and DG.

Emotions and Beliefs	

Ultimatum Game	Dictator Game
Desire to keep as much of the available budget as possible	Desire to keep as much of the available budget as possible

Empathy	Empathy

Fear that too low an offer would be rejected	

Sense of fairness	Sense of fairness

Thinking that it would be convenient for the other participant to accept any offer	Thinking that the other participant could only accept one’s offer

After the EBs assessment, the experiment ended, and the participants were debriefed about its real aims and thanked. The post-experiment interviews confirmed the believability of the procedure: in particular, the participants were informed about deception, but did not express any doubts on the presence of the other player and on the manipulated outcome of the luck game. Moreover, they had believed that after the luck game both participants had some tokens and that the role of the proposer was been randomly assigned. In addition, they had comprised the rules of the UG and the DG. Finally, participants were asked again if they consented to the utilization of the collected data.

**FIGURE 1:**
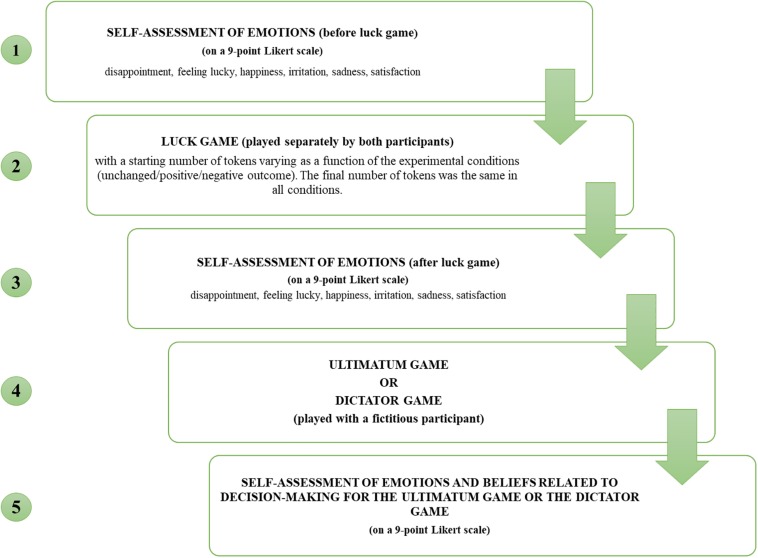
Structure of the study: steps of experimental manipulation.

The procedure is illustrated in Figure 1.

## Results

All data analyses were conducted using SPSS 18.0 IBM software. Data were scrutinized for outliers and four outliers were found. Thus, we performed all statistical analyses with and without outliers. Since the results did not change, we report the analyses conducted on the entire sample.

The first analysis was performed to check whether the manipulated outcome of the card game influenced the intensity of the five emotions that were self-assessed before and after the game.

**FIGURE 2:**
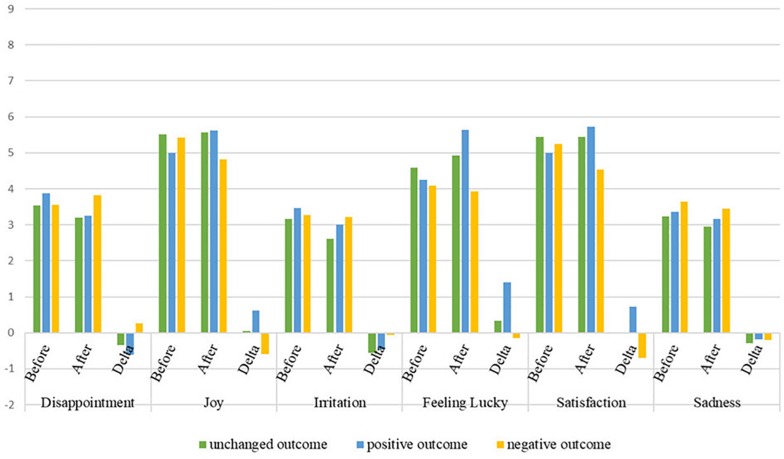
Means of emotions assessed before and after the task manipulation and Δ values as a function of the outcome.

Preliminarily, we calculated the delta (Δ) value (i.e., the post-value minus the pre-value) for each emotion (see Figure 2 for the means of emotions before and after the experimental manipulation and Δ values).

Then a 3 (outcome: unchanged/positive/negative) × 2 (game: UG/DG) between-subject MANCOVA was conducted, with Δ emotions settled as dependent variables and gender included as a covariate (*M* = 1; *F* = 0). The type of game was included in the analysis to check for possible casual effects due to this variable. However, as expected, it did not affect the results. The multivariate tests were significant only for outcome and gender. The univariate tests revealed that outcome affected Δ disappointment, Δ joy, Δ feeling lucky, and Δ satisfaction; gender affected Δ joy, Δ feeling lucky, and Δ satisfaction (the results of MANCOVA are reported in Table 1 in Supplementary Material).

Pairwise comparisons with Bonferroni adjustment showed that Δ disappointment was significantly higher in the negative (NEG-OUT) than in the positive outcome (POS-OUT); Δ feeling lucky was higher in the positive outcome than in both an unchanged outcome (UNC-OUT) and NEG-OUT, which did not differ from each other; finally, Δ joy and Δ satisfaction were highest with POS-OUT, followed by UNC-OUT, and then by NEG-OUT. Females reported higher scores than males on Δ joy, Δ feeling lucky, and Δ satisfaction. As Δ irritation and Δ sadness were not modified by outcome manipulation, they were excluded from further analyses.

**FIGURE 3:**
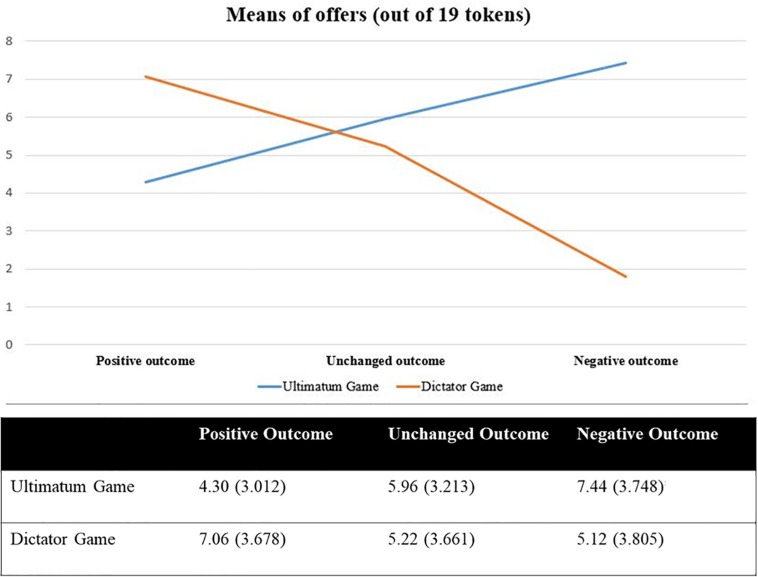
Two-way interaction effect of outcome and game on offer (=number of tokens from 0 to 19) with means (and SD) values as a function of outcome and game.

To investigate whether the manipulated outcome and the type of game influenced, either singularly or in interaction, the participants’ offers, a 3 (outcome) × 2 (game) between-subject ANCOVA was conducted, with gender included as a covariate. The results showed only a significant interaction between game and outcome: *F* = 13.311; df = 2.293; *p* < 0.001; pη^2^ = 0.083 (see Figure 3). The simple effect analysis with Bonferroni adjustment for pairwise comparisons revealed that in the control condition, proposers’ offers did not differ between the two games (*p* = 0.290); with a positive outcome, proposers offered significantly more tokens in the DG than in the UG (*p* < 0.001); conversely, with a negative outcome, proposers gave significantly more tokens in the UG than in the DG (*p* < 0.01).

A similar ANCOVA was performed on the reaction times employed to decide the offer amount. The results revealed only a main effect of the game, *F* = 5.967; df = 1.293; *p* < 0.05; pη^2^ = 0.020, although the pη^2^ value was low. In the UG, participants took a little more time to decide (mean = 38786.6200 s; SD = 1245.726) compared to the DG (mean = 34448.8933 s; SD = 1245.726), a finding compatible with the greater complexity of the UG.

The subsequent analyses were one moderated mediation analysis and two mediation analyses.

The moderated mediation analysis tested whether the effect of the outcome manipulation on offer, moderated by the type of game, as the ANCOVA revealed, was mediated by the changes induced by the experimental manipulation in the four emotions. The analysis was conducted using the PROCESS 3.1 macro for SPSS (Hayes, 2018). The macro employs a bootstrapping method for estimating indirect effects, i.e., the effects of the independent variable (IV) on the dependent variable (DV) through mediator(s): 95% bias-corrected confidence intervals were calculated through 5000 bootstrap samples. We tested a model (The model 5 of Process is depicted in Figure 1, in Supplementary Material), in which the card-game outcome was included as IV; Δ disappointment, Δ joy, Δ feeling lucky, and Δ satisfaction were included as mediators (Meds); and the type of game was included as the moderator (Mod). The offer in the UG and DG was the DV. Gender was included as a covariate.

The multicategorical IV was coded as two dummy variables: POS-OUT and NEG-OUT, with UNC-OUT as a reference category. The Mod was coded as one dummy variable (0 = DG, 1 = UG), as well as the covariate (*M* = 1; *F* = 0).

The results were analogous to those already highlighted by the MANCOVA, as regards the effects exerted by IV on Meds, and to those of the ANCOVA, as regards the interaction effect exerted by IV and Mod on the DV. However, the putative Meds did not exert any direct or indirect effect on offers (the results of the moderated mediation analysis are reported in Table 2 in Supplementary Material)^1^.

**TABLE 2:** Means (and SD) of emotions and beliefs concerning the offer as a function of game and outcome.

	Ultimatum Game			Dictator Game		
		
Emotions and Beliefs	Unchanged Outcome	Positive Outcome	Negative Outcome	Unchanged Outcome	Positive Outcome	Negative Outcome
Desire to keep as much of the available budget as possible	5.02 (2.36)	5.28 (2.67)	3.92 (2.64)	3.26 (2.42)	4.00 (2.52)	3.68 (2.25)
Empathy	5.46 (2.03)	4.54 (2.21)	5.18 (2.34)	4.62 (2.13)	4.98 (2.20)	5.48 (2.48)
Fear that too low an offer would be rejected	3.70 (2.31)	2.78 (2.24)	3.74 (2.60)			
Sense of fairness	5.66 (2.31)	4.76 (2.54)	5.60 (2.49)	4.66 (2.33)	5.28 (2.55)	5.68 (2.57)
Thinking that it would be convenient for the other participant to accept any offer	5.84 (2.36)	5.58 (2.79)	4.18 (2.93)			
Thinking that the other participant could only accept one’s offer				2.78 (2.48)	3.24 (2.79)	2.86 (2.27)

The two mediation analyses were performed to investigate whether the EBs about decision-making mediated the effect of the manipulated outcome on the offer. Since they differed between the games, two mediation analyses were conducted: one for each game. In Table 2 means and SD of EBs are reported by the two games.

**FIGURE 4:**
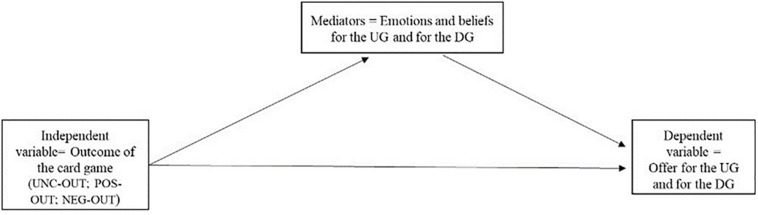
Diagram of the Model 4 – Mediation analysis conducted separately for the UG and the DG.

The first mediation analysis was conducted on the UG, once again using the PROCESS 3.1 macro. We tested a model (model 4, see Figure 4) in which the IV is supposed to affect the DV both directly and indirectly through mediators.

The putative mediators are reported in Table 1. Gender was included as a covariate. Regarding the effect of IV on the putative mediators, the results revealed that, compared to the control condition, the intensity of *empathy* decreased with POS-OUT, whereas the intensity of *desire to keep as much of the available budget as possible* (henceforth: desire to keep for oneself) and *thinking that it would be convenient to the other participant to accept any offer* decreased with NEG-OUT. The other two mediators and gender were not affected by the experimental manipulation.

The total effect (direct + indirect effect) of IV on DV revealed that, compared to UNC-OUT, POS-OUT decreased offers, while NEG-OUT increased them. Gender did not exert any effect. When IV, Meds and the covariate were all inserted into the model to test their direct effects on offer, the results were the following: desire to keep for oneself decreased offers, while *sense of fairness* and *fear that too low an offer would be rejected* increased them. The effects of both positive and negative outcomes were no longer significant, suggesting a full mediation. However, only the effect of desire to keep for oneself mediated the relationship between NEG-OUT and the offer significantly. In particular, NEG-OUT diminished the intensity of this mediator, which in turn diminished the offer. The multiplication of these double negative effects resulted in a positive effect, i.e., receiving a negative outcome in the card game increased offers because it decreased the selfish appropriation. A sense of fairness and fear of rejection increased offers, but this effect did not depend on the manipulated outcome.

The second mediation analysis was performed on the DG, following the same procedure as that for the UG, but with slightly different mediators (see Table 1). Concerning the effect of IV on the putative mediators, the results showed that, compared to the control condition, NEG-OUT increased *empathy* and *sense of fairness*. No other effects were found. The total effect of IV on DV was that, compared to the UNC-OUT, POS-OUT increased offers. When IV, Meds and the covariate were all inserted into the model, the effect of POS-OUT was still significant whereas, among the putative mediators, only desire to keep for oneself, which had not been affected by experimental manipulation, influenced the offer by decreasing it. Therefore, there was no mediation effect. The EBs affected by the experimental manipulation (empathy and sense of fairness) did not affect offer, while the desire to keep for oneself affected offers independently from the experimental manipulation. Gender never affected the results. The results of both mediation analyses are in Tables 3, 4.

**TABLE 3:** Significant results of mediation analysis (Model 4 of Process macro) testing the effects of outcome (IV), emotions and beliefs (mediators), and gender (covariate) on the offer (DV) in the Ultimatum Game.

Ultimatum Game				

Model summary	*R-sq*	*F*	*df*	*p*
	0.382	10.896	8,141	0.000

**Model**	***B***	***t***	***p***	***CI***

Constant	6.050	5.904	0.000	[4.025; 8.077]
Positive outcome	−1.006	−1.703	0.091	[−2.173; 0.162]
Negative outcome	0.889	1.487	0.139	[−0.293; 2.071]
Desire to keep as much of the available budget as possible	−0.600	−5.705	0.000	[−0.807; −0.392]
Empathy	0.016	0.123	0.902	[−0.234; 0.265]
Fear that too low an offer would be rejected	0.238	2.235	0.027	[0.027; 0.449]
Sense of fairness	0.285	2.495	0.014	[0.059; 0.512]
Thinking that it would be convenient for the other participant to accept any offer	0.030	0.324	0.747	[−0.156; 0.217]
Gender	0.308	0.651	0.516	[−0.628; 1.245]

**Total effects of IV on DV (R-sq = 0.131; *F* = 7.345; *df* = 3,146; *p* < 0.001):**				

	***B***	***t***	***p***	***CI***

Constant	5.901	10.678	0.000	[4.809; 6.993]
Positive outcome	−1.660	−2.478	0.014	[−2.984; −0.336]
Negative outcome	1.482	2.212	0.028	[0.158; 2.806]
Gender	0.113	0.207	0.836	[−0.968; 1.195]

**Significant relative indirect effect of IV on DV through:** Desire to keep as much of the available budget as possible				

	***Effect***		***CI***	

Positive outcome	−0.1559		[−0.808; 0.431]	
Negative outcome	0.656		[0.043; 1.400]	

**TABLE 4:** Significant results of mediation analysis (Model 4 of Process macro) testing the effects of outcome (IV), emotions and beliefs (mediators), and gender (covariate) on the offer (DV) in the Dictator Game.

Dictator Game				

Model summary	*R-sq*	*F*	*df*	*p*
	0.170	4.142	7,142	0.000

**Model**	***B***	***t***	***p***	***CI***

Constant	5.703	5.861	0.000	[3.779; 7.626]
Positive outcome	2.082	2.899	0.004	[0.662; 3.501]
Negative outcome	−0.080	−0.110	0.913	[−1.523; 1.362]
Desire to keep as much of the available budget as possible	−0.530	−4.077	0.000	[−0.786; −0.273]
Empathy	0.123	0.867	0.387	[−0.157; 0.403]
Sense of fairness	0.102	0.791	0.430	[−0.153; 0.357]
Thinking that the other participant could only accept one’s offer	0.089	0.717	0.475	[−0.157; 0.335]
Gender	−0.098	−0.166	0.869	[−1.271; 1.075]

**Total effects model of IV on DV (R-sq = 0.059; *F* = 3.063; *df* = 3,146; *p* < 0.05):**				

	***B***	***t***	***p***	***CI***

Constant	5.433	9.098	0.000	[4.253; 6.613]
Positive outcome	1.831	2.460	0.015	[0.360; 3.302]
Negative outcome	−0.035	−0.047	0.963	[−1.516; 1.445]
Gender	−0.462	−0.753	0.453	[−1.675; 0.751]

**There are no significant indirect effects**				

### Statistical Power

We did not determine the sample size *a priori*. However, we conducted a *post hoc* power analysis for each performed analysis through the GPower 3.1 software (Faul et al., 2007) in order to estimate the statistical power of our study. For the ANCOVAs performed on offer and on reaction times, we used the following *a priori* parameters: effect size (f) = 0.25 and α error probability =0.05. The statistical power (1- β) of our sample size was 0.919. For MANCOVA performed on delta emotions, using as *a priori* parameters, effect size f^2^(V) = 0.062 and α error probability =0.05, the statistical power was 0.0997. For the moderated mediation analysis, we used an effect size (f^2^) = 0.15 and α error probability =0.05. The statistical power was =0.999. For the two mediation analyses performed for each game, using the same parameters as the previous analysis, the statistical power was =0.945, for the analysis on the UG, and 0.956 for the one on the DG.

It is noteworthy that a *post hoc* power analysis, as defined by Faul et al. (2007), does not correspond to the retrospective power analysis to calculate the observed power, because in the latter the effect size is estimated from sample data *a posteriori*, whereas in the former it is determined *a priori.*

## Discussion

This study, using a two-stage paradigm, investigated whether a manipulated luck game in the first stage affected offers in the UG and DG and whether this effect was mediated by the changes in the emotions related to the luck game and/or by the EBs about the choice.

The results revealed that the effect of outcome manipulation on offers was moderated by the type of game: a positive outcome increased offers in the DG and decreased them in the UG, whereas with a negative outcome proposers’ offers were higher in the UG than in the DG.

In the control condition, offers did not differ between the two games. Indeed, in this condition, offers in the UG were lower (31.37% of the total budget) than those reported in the literature (40–50%) and thus are similar to those in the DG (27.47%).

Contrarily to our hypothesis, the effects of experimental manipulation were hardly mediated by the changes in EMs or by EBs associated with decision-making. We will discuss this point later.

Before discussing the main result of our study, i.e., the opposite effects that positive vs. negative outcomes have exerted on offers in the UG and DG, it is worth specifying the theoretical and methodological frame in which this result has been obtained and can be interpreted. To this end, it is important to consider the difference between the goals of the present study and those of the studies (e.g., Hoffman et al., 1994; Leliveld et al., 2008; Oxoby and Spraggon, 2008; van Swol and Braun, 2014; Bland et al., 2017; Korenok et al., 2017) that have investigated the relationship between the sense of entitlement or of ownership and offers in the UG and DG. Summing up, previous studies have showed that if proposers feel they have more rights than receivers on the amount size to share, they offer less. This behavior is compatible with the distributive justice, as defined by Aristotle in the Nicomachean Ethics (ca. 340 B.B./English transl. 2011), according to which the available goods should be allocated in conformity with the individual’s merit. The manipulation of the sense of entitlement (deriving from having deserved something) or ownership (deriving from having property rights on something) has tended to induce in the proposers the perception that they have deserved their role by answering better in previous skill tasks (e.g., Hoffman et al., 1994; Oxoby and Spraggon, 2008), that they have contributed more than the other player to the total amount to be shared (e.g., Bland et al., 2017), that they have won at least a part of the endowment to share (van Swol and Braun, 2014), or that the owner of the initial endowment was the player next to whom the endowment was placed (e.g., Leliveld et al., 2008).

In our opinion, however, such manipulations did not clearly distinguish “merit” from “ownership,” and thus, the results of the studies in which they were used did not provide sufficient support for deciding whether proposers offer less because they feel to deserve more, in conformity with the distributive justice, or because they feel to have more property rights on something, irrespective of the source of these rights (see also Hoffman and Spitzer, 1985, and Korenok et al., 2017, for the distinction between the two constructs).

Our study aimed to disentangle “merit” from “ownership” in two ways: (1) by using a luck game, which does not require any personal capacity, to modify the initial endowment of the participants and (2) by making the role of proposer appear as randomly assigned and thus disconnecting it from the results of the luck game played before the UG or DG. In this way, the perception of having deserved something should be ruled out in proposers, while the perception of ownership could be elicited by the simple fact of having at one’s disposal the initial endowment and could be modified by the outcomes of the luck game.

Moreover, it should be stressed that we were interested to examine not only the effects of winnings but also those of losses on offers because our general research question was whether the outcomes of the luck game would lead proposers to make their offers taking into account the difference between the current endowment and the previous one. Put in other words: we were interested in investigating whether good luck or bad luck would foster altruistic or selfish behaviors. In a previous study on the UG (Matarazzo et al., 2017) where this question had been addressed, we only found that a negative outcome decreased offers whilst a positive outcome did not modify them compared to the control condition. Thus, although this finding is compatible with the idea of a general sense of fairness, according to which having less than before authorizes to make less offers, the evidence of the specular behavior had been not found.

Finally, it should be noted that another variation introduced in the present study was that receivers had some endowment: as we have specified in overview, this choice was based on methodological and theoretical reasons. However, we expected that this variation should have reduced offers in both games, compared to those reported in the literature. The control condition, in which the initial budget remained unchanged, has represented the reference point. Actually, only in the UG offers were lower than the average reported in previous studies, whereas in the DG they were similar.

Indeed, the experimental manipulation has had opposite effects for the two games, in line with the third hypothesis we have addressed. The absence/presence of the receivers’ bargaining power is the crucial factor in whose light our results have to be interpreted.

A positive outcome leads the dictators to behave generously and the ultimatum-givers to behave selfishly, whereas a negative outcome leads the latter to increase their offers but does not affect the dictators’ behavior compared to the control condition. Compared to the results of the studies that have manipulated the sense of entitlement and/or of ownership (see section “Introduction”), where offers decreased in both UG and DG when such a sense increased, our findings suggest that, as to the DG, manipulating the outcomes of a luck game produces different effects than manipulating those of a skill task. As we expected, doubling the initial budget due to good luck has not induced in the dictators the belief that they have deserved it and that, consequently, they are authorized to keep most of it for themselves. On the contrary, the DG results suggest that people are willing to donate a non-negligible part (about 37% in our case) of the assets they have won thanks to good luck, provided that they are not constrained to do so, e.g., when the receivers are without bargaining power. In the real world, this behavior is seen when a part of someone’s lottery winnings is given to charity. Interestingly, losing about half of the initial budget in the luck game does not modify the donors’ behavior, compared to the condition in which the budget remains unchanged (about 27% of tokens are given in both conditions). It might be that the receivers’ powerlessness discourages dictators from keeping all or most of their endowment, in conformity with the empathy and sense of fairness that are supposed to underlie cooperative or altruistic behavior (Fehr and Schmidt, 1999, 2006; van Dijk and Vermunt, 2000; Page and Nowak, 2002; Singer et al., 2006; Kirman and Teschl, 2010). In this study, however, although empathy and a sense of fairness increase with a negative outcome, they do not affect the offers. Actually, some features of both the DG’s structure and this specific experiment should have diminished the allocators’ donations: the bargaining powerlessness of receivers, the impossibility to reciprocate the donation, the consideration that receivers have some endowment^2^, and the fact that anonymity, which should reduce offers (Hoffman et al., 1994; Charness and Gneezy, 2008; Franzen and Pointner, 2012), was carefully assured between players and between players and experimenters. Yet, no main effect of the type of game was found: irrespective of the experimental condition, the offers in the DG are overall analogous to those in the UG and higher than the average reported in the literature (Engel, 2011). So, we think that our results are compatible with three explicatory hypotheses. According to the first, dictators behave following a feeling of social responsibility (van Dijk and Vermunt, 2000; Handgraaf et al., 2008). According to the second, offers in the DG are an expression of a non-conscious mandatory duty to donate something when one is in a position to do so, which is probably intrinsic to the Catholic culture. Following this rule, those who have more should give more, and this is what happens with donors in our study. A third hypothesis refers to the theory of warm-glow giving or impure altruism (Andreoni, 1990), according to which giving to others elicits an emotional reward (i.e., satisfaction or warm-glow) that represents the selfish recompense for “altruistic” behavior. All the three hypotheses are compatible with the consideration that receivers are without bargaining power.

Conversely, when receivers have bargaining power, as in the UG, the relationship between the two players changes its configuration and becomes a strategic relationship in which self-interest becomes the prevailing motivation.

From the proposers’ perspective, winnings appear to fuel the sense of ownership, whereas losses seem to induce the opposite effects. Thus, offers decrease with positive outcomes – in conformity with the studies that manipulated the sense of ownership through luck games (van Swol and Braun, 2014; Bland et al., 2017) – and increase with negative outcomes. These results differ from those of our previous study (Matarazzo et al., 2017) in which a similar manipulation was used, with two differences: the role of proposers was not random because after the results of the luck game, participants were told that they have to share their current endowment with the other participant, which had executed a task not entailing any endowment. In the present study, the role of proposers was ostensibly random and receivers had some endowment. The latter information, that seems to have had no effect on dictators’ offers, has conversely played a crucial role in the UG. Seemingly, proposers perceived the receivers as staying on an equal – if not more advantageous – footing because they had both bargaining power and endowment. In our opinion, both factors decreased generosity because receivers were not needy and not depending on proposers. Indeed, all offers, even those in the control condition, were lower than in the literature, as we expected. In conformity with our hypothesis about the role of the receivers’ endowment, it should be noted that fear of rejection, though having a weak but significant effect in increasing offers, had a similar and quite low intensity in all experimental conditions. Plausibly proposers had assumed, in line with our hypothesis, that receivers, having already something, should be more willing to accept even small offers and less willing to punish proposers leaving them with nothing. In fact, the main motivation underlying offers and leading them to diminish was that of keeping as much of the available budget as possible (desire to keep for oneself). This motivation was also the only one that mediated the effect of the experimental manipulation on offers, in particular the one of the negative outcome, a finding that we will discuss later. However, since the intensity of the desire to keep for oneself was similar in both positive and unchanged outcomes, this emotion is unable to explain why offers decreased after a positive outcome, even compared to the control condition. Perhaps the expression “keeping as much of the available budget as possible,” through which we aimed to capture the sense of ownership, was not sufficiently fine-tuned to capture it and/or to distinguish it from other similar constructs, such as a general desire to possession, a wish to preserve one’s self-interests, or a tendency to be greedy.

Concerning the increase of offers after a negative outcome, the findings that fear of rejection did not augment in this condition, whereas the desire to keep for oneself was diminished, disconfirm our hypothesis that higher offers in the UG would have been motivated by fear of rejection and would have represented an expression of risk aversion (Holt and Laury, 2002; Karagözoğlu and Urhan, 2016). On the contrary, our findings suggest that the loss of nearly half of their initial endowment led proposers to be less motivated to save their current endowment and thus more disposed to increase offers, as showed by the mechanism through which the desire to keep for oneself mediated the effect of the negative outcome on offers. To the best of our knowledge, no study has so far highlighted that higher offers can be motivated by a sort of detachment from ownership (or at least from one’s current endowment).

As to our second research question, the results do not corroborate the hypothesis that changes in EMs should mediate the effect of experimental manipulation on offers. Although the manipulated outcome modified the intensity of four of the six emotions we took into account in both the UG and DG, none of them served as mediators. Moreover, none of them had a direct effect on offers.

This finding contrasts with the large body of research sustaining the informational and motivational function of emotion in decision-making (see section “Introduction”) and, more specifically, with a few studies showing the influence of incidental affects on offers in the UG and DG (Mellers et al., 2010; Forgas and Tan, 2013). However, these studies adopted a different paradigm from ours: they induced positive or negative affect through a task that was ostensibly unrelated with UG or DG and then observed the effect of this induced affect, assumed as independent variable, on offers. Furthermore, they used a global measure of emotional reaction, based on its positive/negative valence, and did not examine the effect of specific emotions on decision-making. In our study the experimental manipulation produced changes in four discrete emotions, thus participants played the UG or the DG in emotional states different from the beginning of the experiment. Nevertheless, their decisions were not significantly affected by their emotional experiences. So, emotions echoed the changes in environment, as posited by the functional theories of emotion (e.g., Lazarus, 1991; Lerner and Keltner, 2000; Roseman, 2008; Zeelenberg et al., 2008), but did not motivate decision-making, as stated by the same theories. In our opinion, the information they entailed was motivationally redundant compared to that involved in the behavioral manipulation. This explicative hypothesis has to be further tested, in order to examine whether emotions motivate behavior when cognitive information is lacking or inadequate, when they reach high intensity, or when their source is misattributed, as it happens for incidental emotions (Schwarz and Clore, 2003).

For what regards EBs associated with decision-making, the results slightly differ from those of EMs. Concerning the DG, the negative outcome increased empathy and sense of fairness, but they did not affect offers’ amounts, and thus no mediation effect was found. However, the desire to keep for oneself had a direct effect on offers, by decreasing them, but it was not affected by the experimental manipulation. The finding that “thinking that the other participant could only accept one’s offer” (in which the receivers’ powerlessness was highlighted) has not affected offers and has had a similar and low intensity in all experimental conditions shows that this consideration, which in principle should have allowed dictators to give less, has been not relevant in decision-making. In our opinion, this result further corroborates the idea that the prevailing motivation of the dictators’ offers was to give a gift, since their condition allowed them to do it.

As to the UG, we have already discussed the role of the desire to keep for oneself and fear of rejection on offers. Here we would stress that both fear of rejection and sense of fairness, though not affected by the experimental manipulation, had a weak but significant effect on offers by increasing them. This finding is clear evidence, rather than an inference, that a concern for fairness and fear of rejection are structural features of the UG, and it suggests that they can be co-present rather than mutually exclusive in the ultimatum-givers’ behavior, contrary to the positions frequently expressed in the literature (see section “Introduction”).

Overall, our results suggest that, irrespective of the contextual factors in which UG and DG are played, the structure of these games entails some EBs that directly affect decision-making. In particular, the desire to keep for oneself (which can be viewed as a genuine expression of selfishness) seems to be a fundamental motivation underlying people behavior when they have something to dispose of. From the perspective of the effects of the experimental manipulation and regardless of its incidence on offers, our results highlight that negative outcomes tend to increase moral tendencies whereas positive outcomes tend to decrease them, a finding that echoes the effects of negative and positive mood on social relationships (Forgas and Tan, 2013).

The finding that EBs play a more relevant role in the UG than in the DG is plausibly due to the more complex structure of the former, which is a strategic interaction, due to the receiver’s bargaining power.

In sum, our study has shown that contextual factors, such a manipulated luck game, interact with the different structures of the UG and DG, producing specific and peculiar effects on offers. Conversely, the role of emotional factors appears to be less relevant than in the literature.

A final consideration concerns the legitimacy of using unpaid participants and a non-economic version of both UG and DG, in which the endowment was represented by tokens, not by money. Although one cannot rule out the possibility of an automatic equivalence between tokens and euros, the findings that offers varied as a function of both experimental manipulation and type of game allow us to infer that participants took both the experimental instructions and their endowment seriously, irrespectively of its nature.

## Limitations and Future Directions

The major limitation of this study has been that some constructs, such as the feeling of having property rights on one’s endowment and the tendency to be greedy have not been directly assessed. Maybe, their inclusion among the EBs related to decision-making would have allowed us to distinguish these constructs from the desire to keep for oneself, which in our opinion should have captured the sense of ownership. Consequently, this inclusion should have enabled us to understand the mechanisms through which, in the UG, a positive outcome decreased offers, given that the desire to keep for oneself, the highest motivation underlying the diminution of offers, was similar in both positive and unchanged outcomes.

Possibly, a second limitation is that we did not sufficiently consider how individual differences might interact with experimental manipulation and the type of game. Actually, we have considered some individual differences, such as sense of fairness, empathy, desire to keep for oneself etc., in the EBs related to decision-making. We have found that some of them have been affected by the experimental manipulation or have affected offers, depending on the type of game. However, we did not create different sub-groups of participants, as a function of pre-assessed individual differences, such as fairness concern or level of selfishness, on the basis of measures independent from specific decision-making task.

A further limitation has been that the endowment of both participants at the end of the luck game has not been manipulated. In fact, a way to eliminate the uncertainty about the receivers’ endowment without renouncing to the plausibility of the random assignation of the proposer role would have been to vary the receivers’ endowment along with fixed values (e.g., a half or one quart of the proposers’ endowment). In this way, the role of fear of rejection in the UG would have been assessed more precisely.

Actually, the introduction of this modification in the present study would have augmented the number of experimental conditions too much. Nevertheless, a subsequent study, conducted only on the UG (where these limitations have perhaps affected the results or their interpretability), should take into account these suggestions.

## Data Availability Statement

The data are available in the following repository: https://doi.org/10.6084/m9.figshare.9472334.v1.

## Ethics Statement

The study was approved by the Ethical Committee of the Department to which the corresponding author belongs. Before the experiment, all participants gave their informed consent in accordance with the Declaration of Helsinki. Note that in the request for approval it was specified that the study entailed deception.

## Author Contributions

OM and BP designed the experiments. BP and CG implemented the experiments and conducted the pilot study. BP collected the data with the contribution of CG. OM and BP conducted the statistical analyses. BP wrote the first draft of the manuscript. OM wrote the final manuscript. CG prepared the tables, Supplementary Material, and references. All authors approved the submitted version of the manuscript.

## Conflict of Interest

The authors declare that the research was conducted in the absence of any commercial or financial relationships that could be construed as a potential conflict of interest.

## Footnotes

1 A factor analysis was performed on delta EMs to reduce their number. Three main components were found: negative emotions, positive emotions, and feeling lucky. Then, a MANCOVA and a moderated mediation analysis were conducted on factorial scores, but the results were analogous to the ones with all six emotions. Thus, we did not report the results on the factorial scores.

2 Note that the only study, to our knowledge, that has found that offers increase when the receivers’ endowment is uncertain (Engel and Goerg, 2018) also shows that “dictators do not care about the whole endowment distribution, but about the possibility of the recipient ending up with nothing” (p. 60).
