# Supplementary material for: Influences of a Luck Game on Offers in Ultimatum and Dictator Games: Is There a Mediation of Emotions?
Source: Front Psychol. 2020 Feb 3;11:13. doi: 10.3389/fpsyg.2020.00013 (PMC7008780; doi:10.3389/fpsyg.2020.00013)

Supplementary material

Table 1 – Multivariate and univariate tests of MANCOVA performed on delta (Δ) emotions (the post-value less the pre-value).

**Multivariate tests:**	**Pillai's Trace**	**df**	**F**	**p**	η_p^2
Intercept	.126	6,288	6.940	.000	.126
Outcome	.217	12,578	5.849	.000	.108
Game	.032	6,288	1.569	.156	.032
Outcome x Game	.032	12,578	0.772	.680	.016
Gender	.068	6,288	3.508	.002	.068
**Univariate tests :**	**Dependent Variable**	**df**	**F**	**p**	η_p^2
Outcome	Δ Disappointment	2	4.419	.013	.029
	Δ Feeling Lucky	2	13.601	.000	.085
	Δ Irritation	2	1.962	.142	.013
	Δ Joy	2	16.374	.000	.101
	Δ Sadness	2	.124	.883	.001
	Δ Satisfaction	2	13.761	.000	.086
Game	Δ Disappointment	1	.358	.550	.001
	Δ Feeling Lucky	1	3.241	.073	.011
	Δ Irritation	1	.127	.721	.000
	Δ Joy	1	.025	.874	.000
	Δ Sadness	1	2.232	.136	.008
	Δ Satisfaction	1	1.833	.177	.006
Outcome x Game	Δ Disappointment	2	.675	.510	.005
	Δ Feeling Lucky	2	1.139	.321	.008
	Δ Irritation	2	.245	.783	.002
	Δ Joy	2	.430	.651	.003
	Δ Sadness	2	.628	.534	.004
	Δ Satisfaction	2	1.373	.255	.009
Gender	Δ Disappointment	1	.166	.684	.001
	Δ Feeling Lucky	1	7.222	.008	.024
	Δ Irritation	1	.423	.516	.001
	Δ Joy	1	8.977	.003	.030
	Δ Sadness	1	.072	.789	.000
	Δ Satisfaction	1	10.943	.001	.036
df error		293			

Table 2 – Significant results of the moderated mediation analysis (Model 5 of Process macro) testing the effects of Outcome (IV), Delta emotions (mediators), Game (moderator) and gender (covariate) on the offer.

Model summary	***R-sq***	***F***	***df***	***p***
	.111	3.598	10,289	.000

Model	***B***	***t***	***p***	***CI***
Constant	5.360	9.882	.000	[4.293; 6.428]
Positive outcome	1.977	2.775	.006	[.575; 3.379]
Negative outcome	-.235	-.327	.744	[-1.650; 1.181]
Δ Disappointment	-.056	-.568	.570	[-.249; .137]
Δ Feeling Lucky	-.100	-1.005	.316	[-.297; .096]
Δ Joy	-.282	-1.939	.054	[-.569; .004]
Δ Satisfaction	.126	1.110	.268	[-.097; .349]
Game	.795	1.125	.262	[-.596; 2.186]
Positive outcome x Game	-3.455	-3.453	.001	[-5.425; -1.486]
Negative outcome x Game	1.665	1.656	.099	[-.314; 3.643]
Gender	-.286	-.674	.501	[-1.119; .548]

Conditional effect of the focal predictor at values of the moderator:

Outcome	*Game*	*Effect*	*t*	*p*	*CI*
Positive	DG	1.977	2.775	.006	[.574; 3.379]
Negative	DG	-.235	-.327	.744	[-1.650; 1.180]
Positive	UG	-1.479	-2.035	.043	[-2.908; -.049]
Negative	UG	1.430	1.993	.047	[.018; 2.842]

There are no significant indirect effects

Figure 1 – Diagram of the Model 5 – Moderated mediation analysis

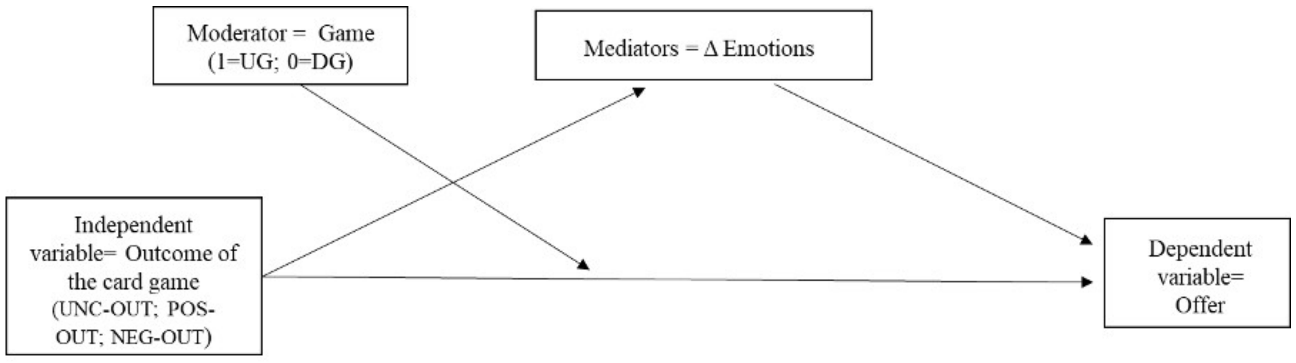

Supplement: Supplementary file 1 [file Data_Sheet_1.pdf]
